# The regulation of T helper cell polarization by the diterpenoid fraction of *Rhododendron molle* based on the JAK/STAT signaling pathway

**DOI:** 10.3389/fphar.2022.1039441

**Published:** 2022-10-25

**Authors:** JiaXu Qin, XiangWei Zheng, YanChen He, Yang Hong, Shuang Liang, Xin Fang

**Affiliations:** Engineering Research Center of Modern Preparation Technology of Traditional Chinese Medicine, Ministry of Education, Innovation Research Institute of Traditional Chinese Medicine, Shanghai University of Traditional Chinese Medicine, Shanghai, China

**Keywords:** *Rhododendron molle*, diterpenoid fraction, polarization, Th cells, JAK/STAT signaling pathway

## Abstract

The diterpenoid fraction (DF) prepared from fruit of *Rhododendron molle* was shown to have potential therapeutic effects on collagen-induced arthritis (CIA) rats based on our previous studies. As a continuation of those studies, herein, a lipopolysaccharide-induced endotoxin shock mouse model was used. The results showed that 0.2 mg/ml of DF significantly increased the mouse survival rate and had an anti-inflammatory effect. Further studies showed that DF could decrease the proportion of T helper cells (Th1 and Th17), and increase the proportion of Th2 and regulatory T cells (Tregs). Enzyme-linked immunosorbent assays indicated that DF inhibited the secretion of inflammatory cytokines such as TNF-α, IL-1β, and IL-6; western blotting showed that DF significantly reduced the levels of phosphorylated STAT1 and STAT3. *In vitro*, DF could dose-dependently inhibit the polarization of naive CD4^+^ T cells to Th1 or Th17 cells. DF at 10 μg/ml could markedly decrease the expression of mRNA encoding IFN-*γ* and T-bet, and suppress Th1 differentiation by downregulation of the activity of STAT1 and STAT4. Meanwhile, DF at 10 μg/ml remarkably reduced the expression of mRNA encoding IL-17a, IL-17f, and ROR*γ*t, and downregulated STAT3 phosphorylation, suggesting that DF could inhibit Th17 differentiation by reducing STAT3 activation. Taken together, DF blocked the JAK/STAT signaling pathway by inhibiting STAT1 and STAT3 phosphorylation, which clarified the important role of JAK/STAT signaling pathway in anti-rheumatoid arthritis.

## 1 Introduction

*Rhododendron molle* G. Don, a Rhododendron genera plant of the Ericaceae family, is recorded as a toxic Chinese herbal medicine in Sheng Nong’s herbal classic, and in folk medicine it is used to treat rheumatoid arthritis (RA) (JiangsuNew Medical College, 1986; Luo et al., 1993; Guan and Liu, 2002). For its further development, we established a process to prepare the diterpenoid fraction (DF) from *R. molle* fruit using resin and silica gel chromatography methods, and evaluated the antinociceptive activity, mechanism of action and toxicity of Rhodojaponin III, the main component of DF (Yao et al., 2019; Yang et al., 2022). On the one hand, *in vivo* experiments indicated that DF had therapeutic effects on a collagen-induced arthritis (CIA) rat model and exerted analgesic and anti-inflammatory effects. On the other hand, *in vitro* experiments showed that DF had significantly inhibited the proliferation of T and B lymphocytes (He et al., 2021). Preliminary mechanistic studies suggested that the anti-RA effects of DF were related to its immune function and inhibition of tumor necrosis factor (TNF)-α and pro-inflammatory cytokines, e.g., interleukin (IL)-6 and IL-1β (He et al., 2021). Rheumatoid arthritis is an autoimmune disease, and in the immune response to RA, the major players are CD4^+^ T-helper (Th) lymphocyte subsets (Boniface et al., 2013). Th1, Th2, and pro-inflammatory Th17 cell numbers are increased (Aeberli et al., 2005; Yang et al., 2004), while those of regulatory T cells (Tregs) are decreased in the peripheral blood of patients with RA (Chavele and Ehrenstein, 2011). Therefore, medicines targeting the differentiation of Th1 and Th17 cells might represent promising candidates in the treatment of autoimmune diseases (Cui et al., 2009). The critical role of JAK/STAT pathway activation in RA was further confirmed by the US Food and Drug Administration (FDA) approval of the JAK3 selective small molecule inhibitor (SMI), tofacitinib, for the pharmacological treatment of RA (Ciobanu et al., 2020). Indeed, various natural compounds that have been proven to be beneficial in attenuating inflammation in RA act via modulation of the JAK/STAT signaling pathway (Kour et al., 2022). In addition, IL-6 can predominantly activate JAK/STAT (Vieira et al., 2016). IL-6 induces STAT1 and STAT3 phosphorylation via the activity of JAK1 and JAK2 (Mihara et al., 2012; Sheppard et al., 2017). Therefore, we studied DF’s effects on the differentiation of Th1 and Th17 cells by inhibiting JAK/STAT signaling both *in vitro* and *in vivo* to further determine the mechanism by which DF ameliorates RA.

## 2 Materials and methods

### 2.1 LPS-induced endotoxin shock model preparation and experiment protocols

#### 2.1.1 Animals

Male C57BL/6 mice (18–20 g, 6–8 weeks old) were provided by Shanghai SLAC Laboratory Animals Co., Ltd. (Shanghai, China), and housed in Fujian Medical University. The mice were raised in specific pathogen-free conditions (22°C and a 12 h light/dark cycle). All animal experiments were conducted in strict accordance with the guidelines of the NIH, and the experimental procedures were approved by the Ethics Committee of Fujian Medical University (FJMU IACUC 2019-0056).

#### 2.1.2 Reagents and chemicals

Chloral hydrate, sodium chloride injection, normal saline, and methanol were bought from Sinopharm Chemical Reagent Co., Ltd. (Shanghai, China). Glycine, TRIS buffer, and sodium dodecyl sulfate (SDS) was purchased from Sangon Biotech (Shanghai) Co., Ltd. (Shanghai, China). Skim milk powder was purchased from BBI Life Sciences (Shanghai, China). 30% acrylamide and ammonium persulfate (AP) were bought from Beijing Dingguo Changsheng Biotechnology Co., Ltd. (Beijing, China). Tetramethylethylenediamine (TEMED) was provided by Sigma (St. Louis, MO, United States). GeneView (Ste. Genevieve, MO, United States) provided the enhanced chemiluminescence (ECL) Kit. The Milli-Q (18.2 MΩ) ultra-pure water system (Millipore, Billerica, MA, United States) was used to prepare pure water. Mouse serum interferon gamma (IFN-γ), IL-10, IL-12p70, IL-17A, TNF-α, IL-6, monocyte chemoattractant protein-1 (MCP1), and IL-1β enzyme-linked immunosorbent assay (ELISA) kits were purchased from Bender (Gruenberg, Germany).

DF was prepared in our laboratory [Batch No.: 20180522. The DF had a rhodojaponin III content of 41.6% and a rhodojaponin VI content of 13.6%, as determined by the linear curve method (Yao et al., 2019)]. Dexamethasone was purchased from GlpBio (Batch No.: GC40775. Montclair, CA, United States).

#### 2.1.3 Experimental protocols

Fifty C57BL/6 mice were randomly divided into five groups, including the blank group, the model group, the DF treatment groups (high and low dose), and the positive control group (*n* = 10 mice per group). DF was intraperitoneally injected at 0.2 mg/kg and 1.0 mg/kg in the treatment groups. The blank group was given the same dose of normal saline, and the positive control group was given 1.0 mg/kg of dexamethasone. Lipopolysaccharide (LPS; 15 mg/kg) was intraperitoneally injected into the model, DF treatment, and positive control groups. The survival rate of the mice within 5 days was observed and recorded every 12 h (Liu et al., 2018).

#### 2.1.4 Histological analysis

Cervical dislocation was used to sacrifice the mice, whose liver, lungs, and kidneys were removed, 4% paraformaldehyde-fixed, dehydrated through an ethanol concentration gradient, paraffin embedded, sectioned, and hematoxylin-eosin (HE) stained. The histopathological changes in the liver, kidneys, and lungs were observed under a microscope and photographed.

#### 2.1.5 Spleen cell proliferation assay

The spleens of the mice in each group were dissected out and rinsed using pre-cooled phosphate-buffered saline (PBS, pH7.4; Gibco, Grand Island, NY, United States). After centrifugation at 4°C for 5 min × 300 *g*, the pelleted spleen cells were suspended in Roswell Park Memorial Institute (RPMI) 1640 containing 10% fetal calf serum (FCS). 100 μL aliquots of the suspended cells (2×10^6^ cells/mL) were added to the wells of a 96-well plate. Half of the wells were added with LPS solution for specific induction, and 100 μL of culture medium was added to the negative control as the proliferation background. In the blank control group, only 200 μL of medium was added. The 96-well plates were placed in an incubator at 37°C with 5% CO_2_ for 24 h. The cells were collected and co-stained of phycoerythrin (PE)-CD3, fluorescein isothiocyanate (FITC)-CD4, and Peridinin chlorophyll protein complex (PerCP)/cyanine (CY)5.5-CD8 (Ebioscience Inc., San Diego, CA, United States) with the CFDA-SE (Carboxyfluorescein diacetate, succinimidyl ester), respectively. The percentages of CD3^+^ T, CD4^+^ T, and CD8^+^ T cells were detected using flow cytometry in a fluorescence activated cell sorting (FACS) Calibur instrument (BD Biosciences, San Jose, CA, United States) (Sun et al., 2012).

#### 2.1.6 Assay of the proportion of Th cells in the spleen

Spleen cell suspension (1 ml) was added to the wells of a 24-well plate at 2×10^6^ cells/well, and 1 ml of LPS solution was added to each well for stimulation. The plates were placed in an incubator at 37°C with 5% CO_2_ for 24 h. Next, 100 ng/ml IL-2 was used to stimulate the spleen cells for 48 h, followed by incubation with 100 ng/ml phorbol 12-myristate 13-acetate (PMA) (Sigma), 750 ng/ml ionomycin, and 2 μm monensin for 5 h. Intracellular staining was then performed. Collected spleen cells were cultured in the presence of anti-CD4 antibodies, fixed, infiltrated, and stained using a mouse Th1/Th2/Th17/Treg phenotype kit (BD Biosciences, San Jose, CA, United States). The cells were analyzed by flow cytometry as in Section 2.1.5.

#### 2.1.7 Measurement of Th cell cytokines

Venous blood samples (1.0 ml) were taken from the mouse’s left external jugular vein at 2×10^6^ cells/mL. Serum IFN-γ, IL-10, IL-12p70, IL-17A, TNF-α, IL-6, MCP1, and IL-1β production was determined using ELISA, as previously described (Hagiwara et al., 2008).

#### 2.1.8 Western blotting

Spleen tissue was incubated with LPS for 24 h, then protein extracts were prepared and processed for western blotting analysis according to a previously published method (Hagiwara et al., 2008). Briefly, proteins on membranes were reacted with primary antibodies at room temperature for 1 h, washed thrice, and then reacted for 1 h with the secondary antibodies. The blots were then washed thrice and developed and exposed according to the instructions of the Odyssey infrared imaging system (BIO-RAD, Hercules, CA, United States).

### 2.2 Effect of DF on the polarization of Th cells and the JAK/STAT pathway *in vitro*

#### 2.2.1 Cells

Spleen cells were isolated from C57BL/6 mice. A single cell suspension of spleen cells was prepared and purified using density gradient centrifugation with Percoll (GE Healthcare, Chicago, IL, United States). A mouse CD4^+^ negative selection kit (Miltenyi Biotec, Bergisch Gladbach, Germany) was employed to remove non-CD4^+^ T cells, and a fluorescence activated cell sorter was used to determine the purity of the spleen cells. The concentration of the isolated mouse CD4^+^ T cells was higher than 80%.

#### 2.2.2 Cell culture

Mouse CD4^+^ T cells (2×10^6^ cells/mL) were cultured in RPMI 1640 medium containing 10% fetal bovine serum and anti-CD3/28 antibody (2 μg/ml, Peprotech, Rocky Hill, NJ, United States) for the stimulation of T cell receptors. We added a Th1 mixture (10 ng/ml IL-12 and 20 μg/ml α-IL-4 antibody, Peprotech) or a Th17 mixture (10 ng/ml IL-6, 10 ng/ml α-transforming growth factor (TGF)-β, 10 ng/ml IL-23, 10 μg/ml α-IL-4 antibody, and 10 μg/ml IFN-*γ* antibody, Peprotech), respectively, for 4 days to drive Th1 or Th17 polarization. Intervention was carried out in the cell culture medium by adding 1 μm fludarabine or HO-3867 (Selleck Chemicals, Houston, TX, United States).

#### 2.2.3 Quantitative real-time PCR reverse transcription PCR

RNAiso Plus (Takara, Dalian, China) was employed to isolate total RNA from spleen cells following the manufacturer’s guidelines. Total RNA (1 μg) was converted to cDNA via reverse transcription using an oligo (dT) primer (Sangon Biotech, Shanghai, China) and the PrimeScript^™^-RT-Master-Mix (Takara). The quantitative real time PCR (qPCR) step of the qRT-PCR protocol was performed using the cDNA as the template with SYBR Premix ExTaq^™^ (Takara). The sequences of the qPCR primers are shown in Supplementary Material.

#### 2.2.4 Flow cytometry

Spleen cells were stimulated for 48 h using 100 ng/ml IL-2, followed by 2 μM Monensin (BD Biosciences), 750 ng/ml ionomycin (Sigma), and 100 ng/ml PMA (Sigma) for 5 h. Collected spleen cells were cultured with anti-CD4 antibodies, fixed, permeabilized, and then stained using mouse Th1/Th17 phenotype kits (BD Biosciences) and FITC-labeled anti-forkhead O3 (FOXP3) antibodies (eBioscience). The FACS Calibur instrument (BD) was used to analyze the stained cells.

#### 2.2.5 Western blotting

Radioimmunoprecipitation assay (RIPA) buffer (Beyotime Biotechnology, Jiangsu, China) was used to lyse the cells, and a Bradford kit (Beyotime Biotechnology) was used to determine the lysate protein concentration. Processing of the membranes proceeded according to the method detailed in Section 2.1.8.

### 2.3 Statistical analysis

GraphPad Prism 7 for Windows (GraphPad Software Inc., La Jolla, CA, United States) was used to analyze the data. The mean ± SD was used to express the quantitative data. To determine the statistical differences between groups, one-way analysis of variance followed by Dunnett’s t test was carried out. *p* values <0.05 were considered statistically significant.

## 3 Results

### 3.1 Anti-inflammatory effect of DF on LPS-induced endotoxin shock model mice

#### 3.1.1 Effect of DF on the survival rate

**FIGURE 1:**
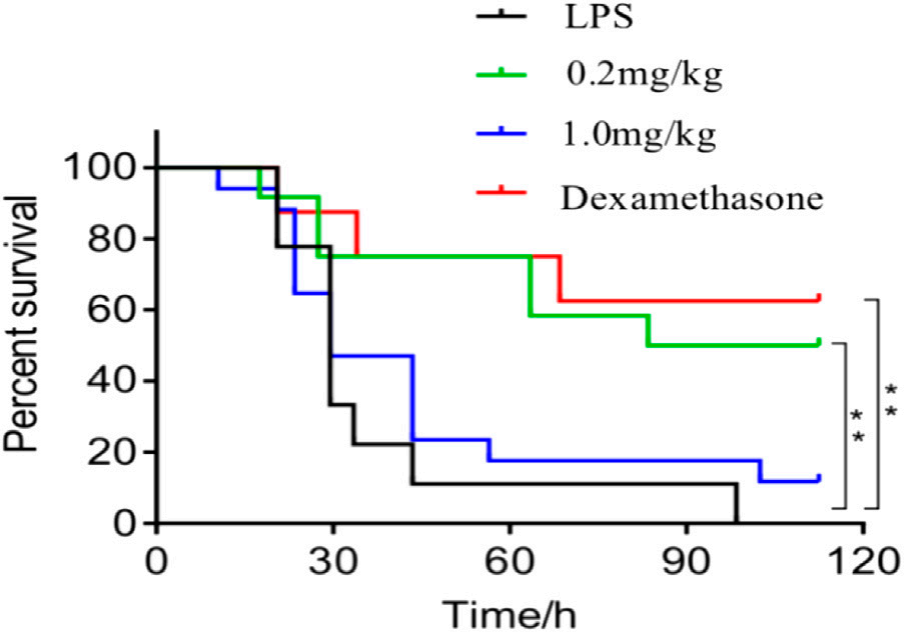
Survival curve of the mice in each group. **, *p* < 0.05, compared with model group.

As shown in Figure 1, the survival rate of the 0.2 mg/kg dose group was 50% within 120 h after treatment with DF, which was similar to the positive control group, indicating that 0.2 mg/kg DF could effectively improve the survival status of the LPS-induced endotoxin shock model mice.

#### 3.1.2 Effects of DF on organ injury

**FIGURE 2:**
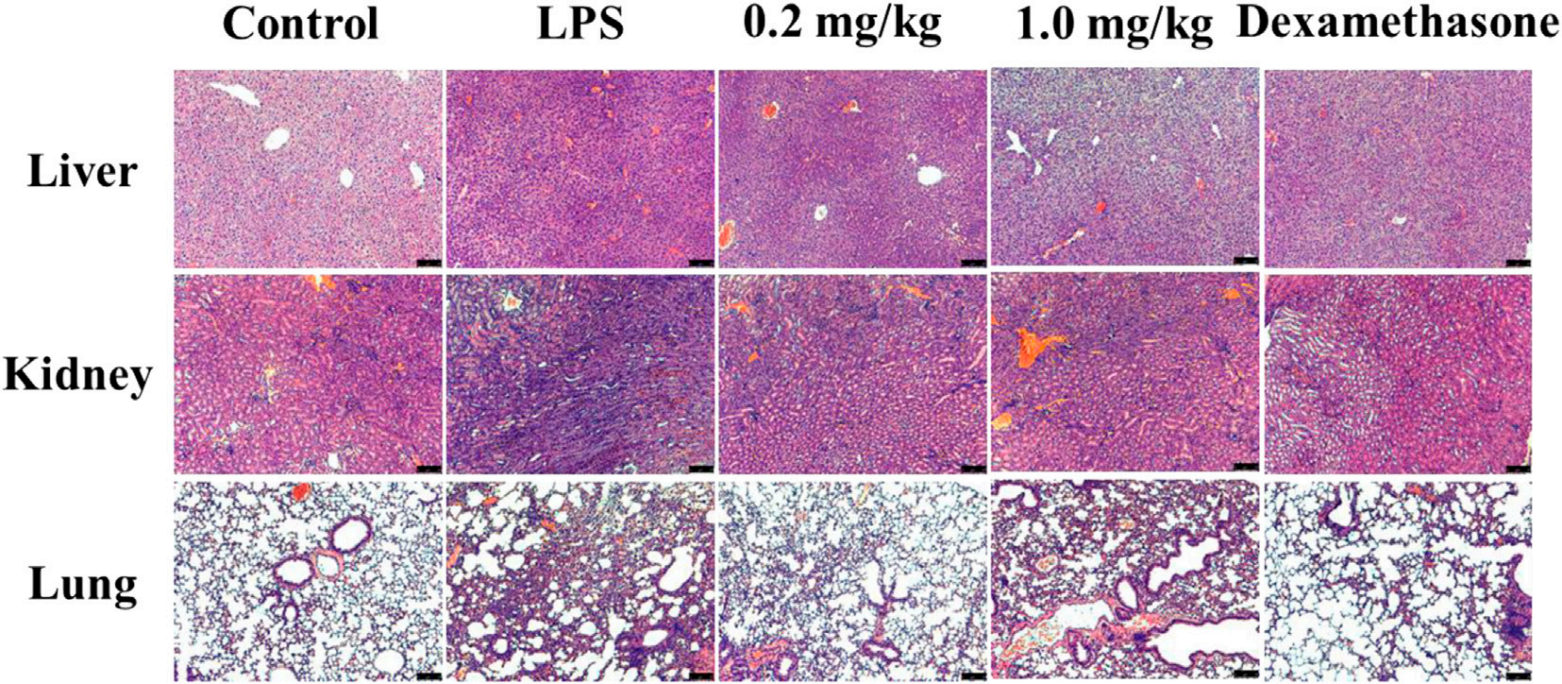
Organ injury of the mice in each group.

Histological analysis showed that severe inflammation occurred in the liver cells of the model group, while a small amount of diffuse necrosis occurred in the livers of the 0.2 mg/kg DF dose group. In the model mice, hyperemia and edema of the adrenal cortex were observed; whereas the renal bleeding and edema were significantly improved in the 0.2 mg/kg DF dose group. In the model group, there was obvious pulmonary capillary hemorrhage, microthrombosis outside the vascular lumen, the alveolar spaces were widened and thickened, some alveolar atrophy occurred, and a large number of inflammatory cells were infiltrated in the alveolar space. In the 0.2 mg/kg DF group, a small amount of red blood cells and inflammatory exudate in the alveolar wall could be found in the lung and vascular lumen, and mild to moderate levels of inflammatory cells were also observed to have infiltrated according to the red pigments (Figure 2).

#### 3.1.3 Effect of DF on the proliferation of spleen cells

**FIGURE 3:**
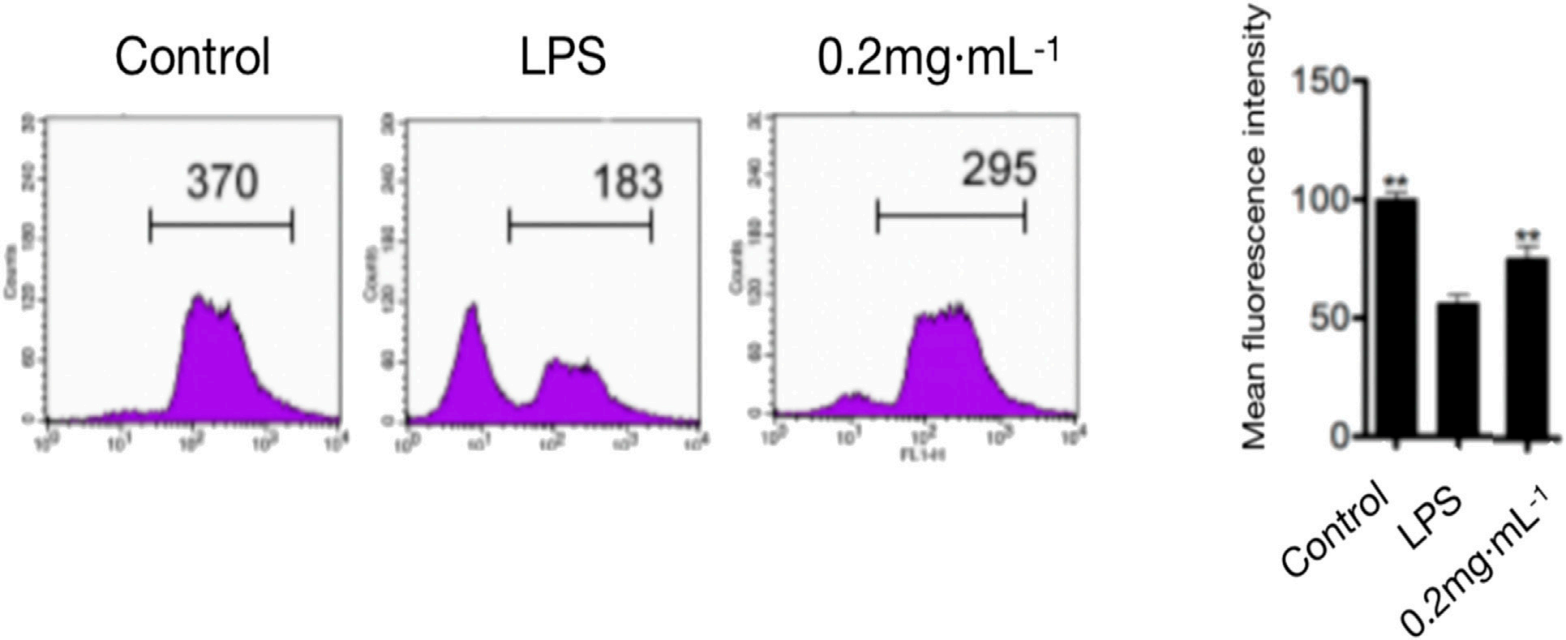
Spleen cell proliferation in mice.

As shown in Figure 3, compared with that in the model group, the 0.2 mg/kg DF dose significantly inhibited LPS-induced lymphocyte proliferation, with an inhibition rate of about 80% (*p* < 0.05).

#### 3.1.4 Effects of DF on the Th cell proportion

**FIGURE 4:**
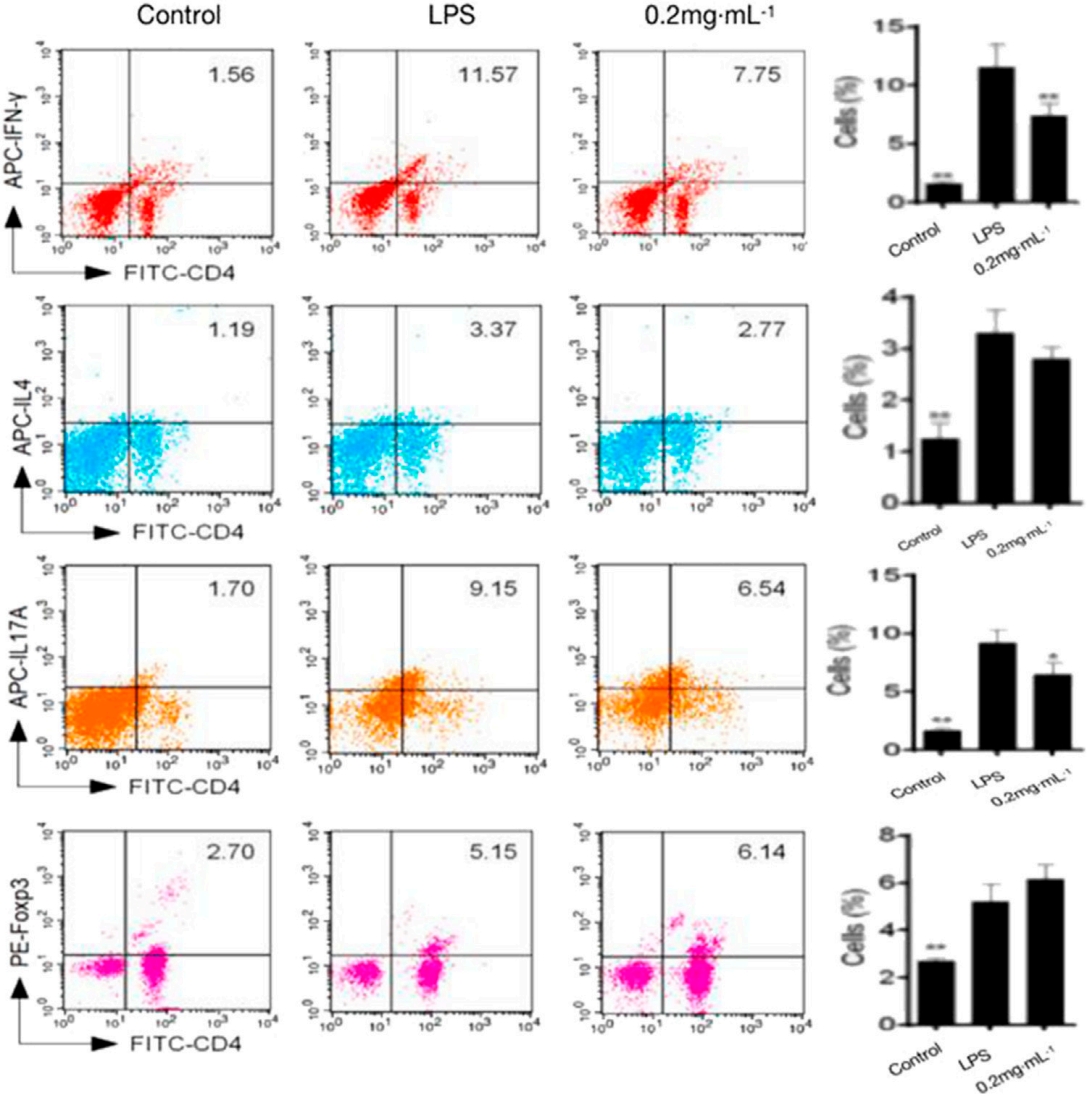
Effects of DF on the proportion of Th cells.

The proportion of inflammatory Th1 and Th17 cells was significantly decreased by DF treatment in comparison with that in the model group; by contrast, the Th2 and Treg proportions increased significantly (Figure 4). The results indicated that *in vivo* administration of DF could reduce the proportions of pro-inflammatory cells and promoted the ratio of Th2 and Treg anti-inflammatory cells.

#### 3.1.5 Effects of DF on Th cell cytokines

**FIGURE 5:**
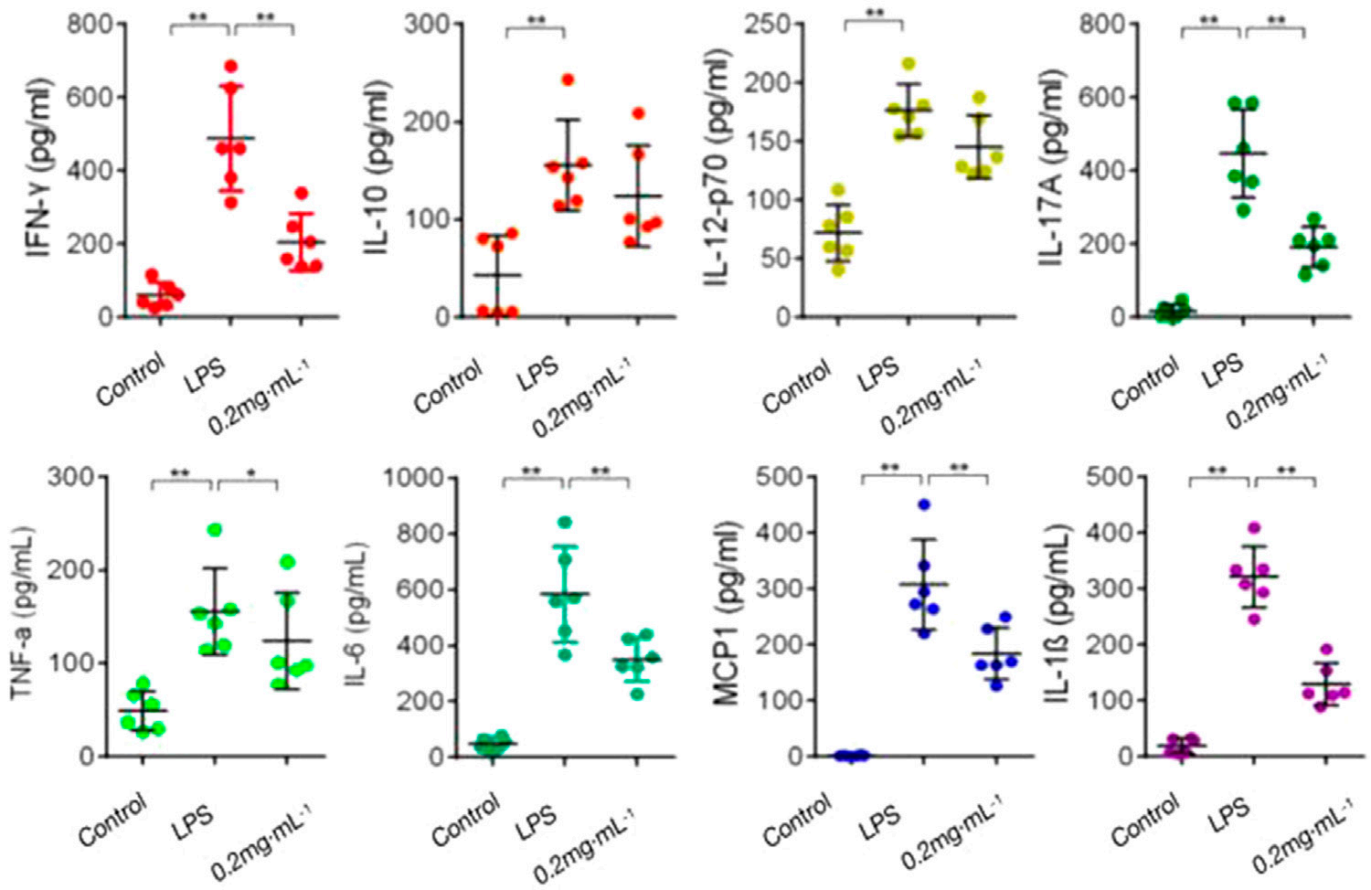
Effect of DF on Th cytokines. **, *p* < 0.01, compared with model group.

The levels of pro-inflammatory cytokines (TNF-α, IFN-γ, IL-1β, IL-6, and IL-17A) and anti-inflammatory cytokine IL-10 in the model group were increased significantly in comparison with those in the blank group. In comparison with those in the model group, pro-inflammatory cytokines (TNF-α, IFN-γ, IL-1β, IL-6, and IL-17A) secretion was significantly inhibited after treatment with DF; however, the effect of DF on IL-10 was not obvious (Figure 5). The results showed that DF could inhibit the secretion of inflammatory cytokines to inhibit the immune response.

#### 3.1.6 Effects of DF on JAK/STAT signaling proteins

**FIGURE 6:**
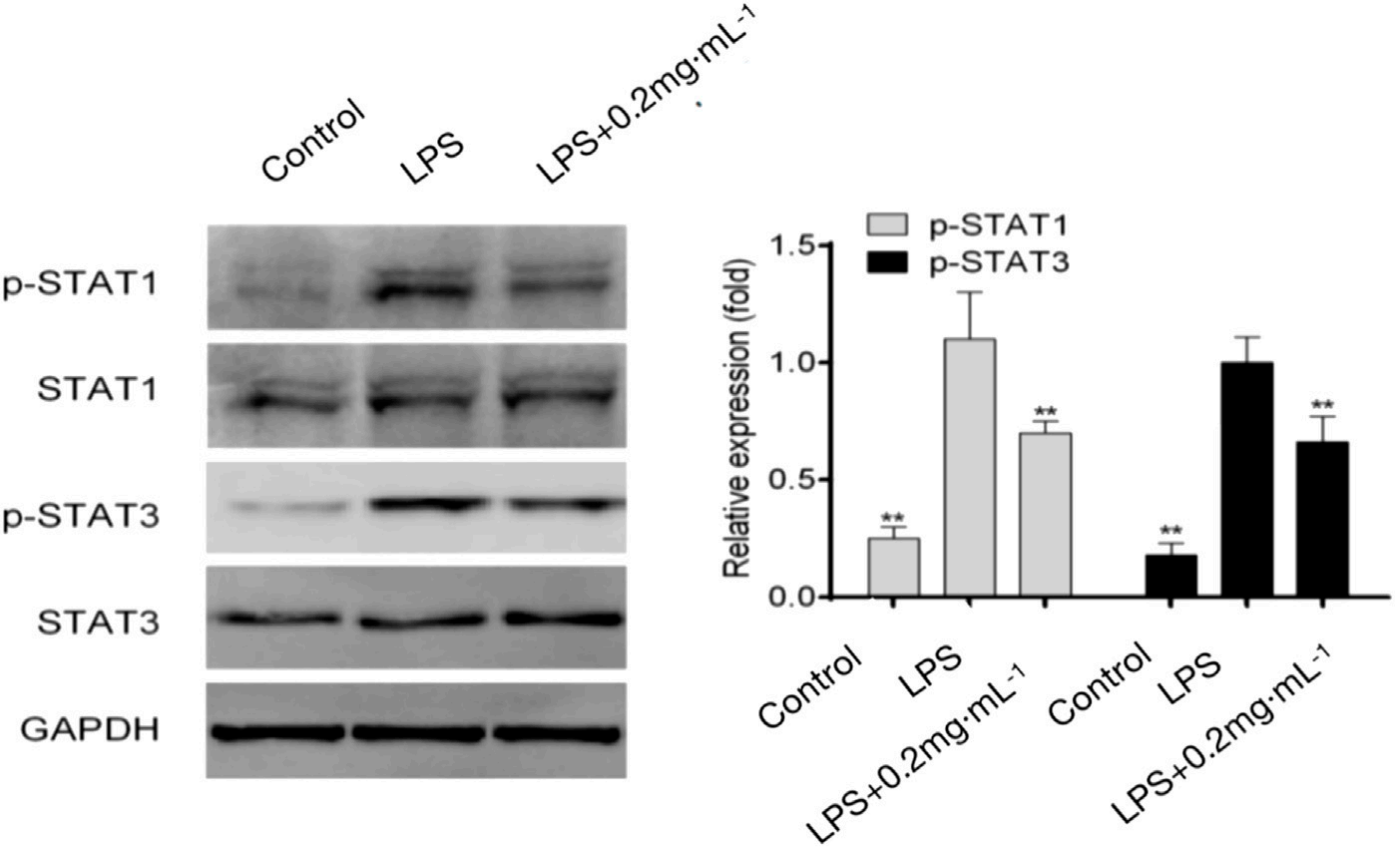
Effect of DF on Th-related STAT proteins. **, *p* < 0.05, compared with model group.

The results showed that JAK/STAT signaling proteins were not significantly phosphorylated in the blank group. The phosphorylation of STAT1 increased significantly in the model group; the combination of STAT1 and IFN-γ induced T cells to differentiate into Th1 cells. The level of Th17-related phosphorylated STAT3 was also significantly increased in the model group. Phosphorylated STAT3 binds to IL-17 and induces the polarization of T cells to Th17 cells. Meanwhile, the 0.2 mg/kg DF dose group showed significant inhibitory effects on STAT1 and STAT3 activation, indicating that DF not only inhibited the phosphorylation of STAT1 to induce Th1 differentiation, but also significantly inhibited the phosphorylation of STAT3 to induce Th17 differentiation (Figure 6).

### 3.2 Effects of DF on the polarization of Th cells *in vitro*

#### 3.2.1 DF suppressed Th1 differentiation by downregulation of the STAT1 and STAT4 signaling pathway

**FIGURE 7:**
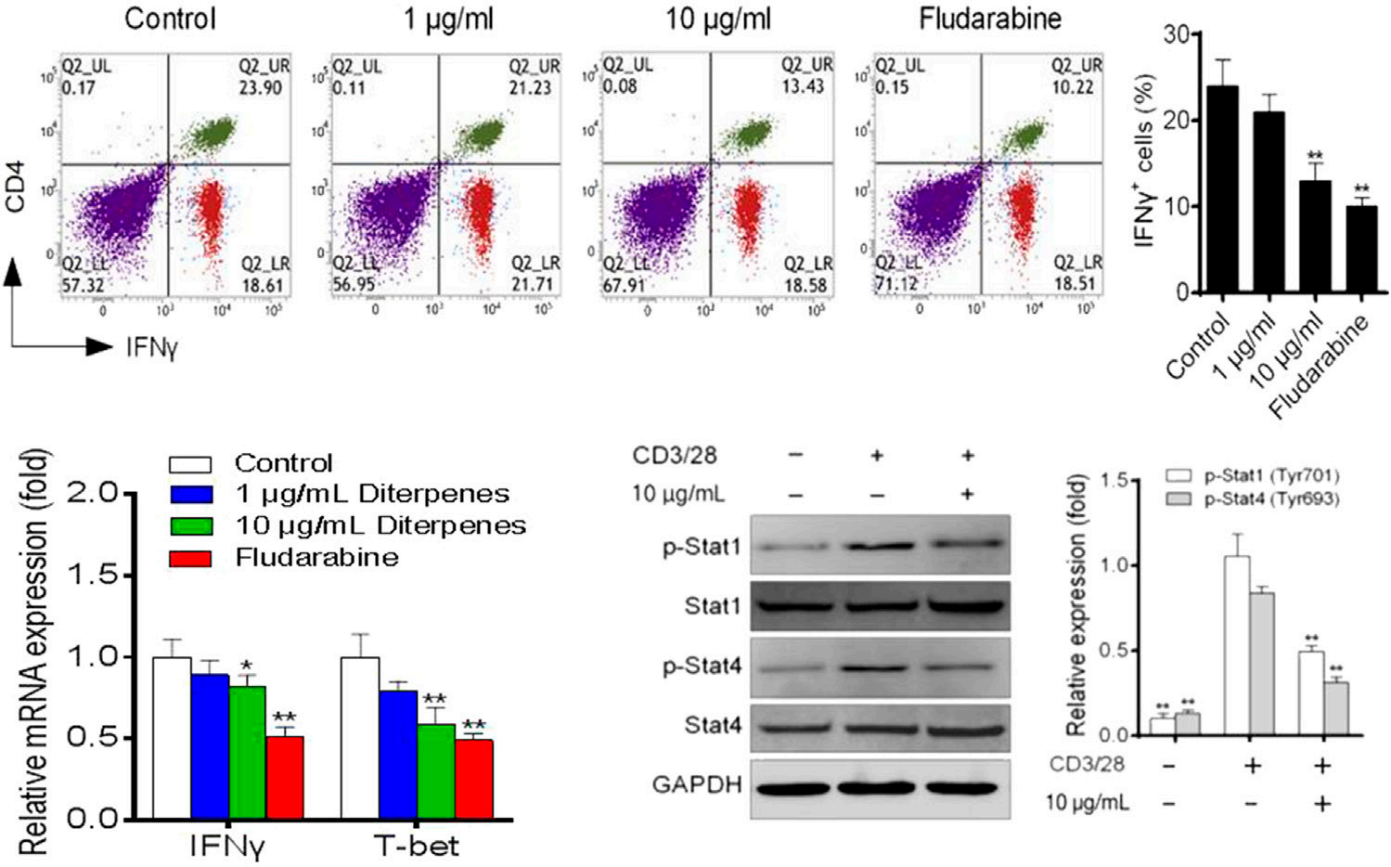
Effect of DF on the proportion of Th1 polarized cells, mRNA levels of Th1 transcription factors, and Th1-related STAT proteins.

Approximately 40% of CD4^+^ T cells differentiated into Th1 cells under induction by the Th1 differentiation mixture (Figure 7). Further experiments showed that, in a dose-dependent manner, DF decreased the proportion of Th1 cells, but did not inhibit the CD4^+^ T cell proliferation and activity. Next, we examined the levels of IFN-*γ* and T-bet markers in Th1 cells. As expected, DF downregulated the mRNA expression of both genes (Figure 7). We further investigated the STAT signaling pathway. Interestingly, 10 μg/ml of DF also decreased the levels of phosphorylated STAT1 and STAT4, indicating that DF reduced STAT1 and STAT4 activation to inhibit the differentiation of Th1 cells (Figure 7).

#### 3.2.2 DF suppresses Th17 differentiation by downregulating STAT3 signaling

**FIGURE 8:**
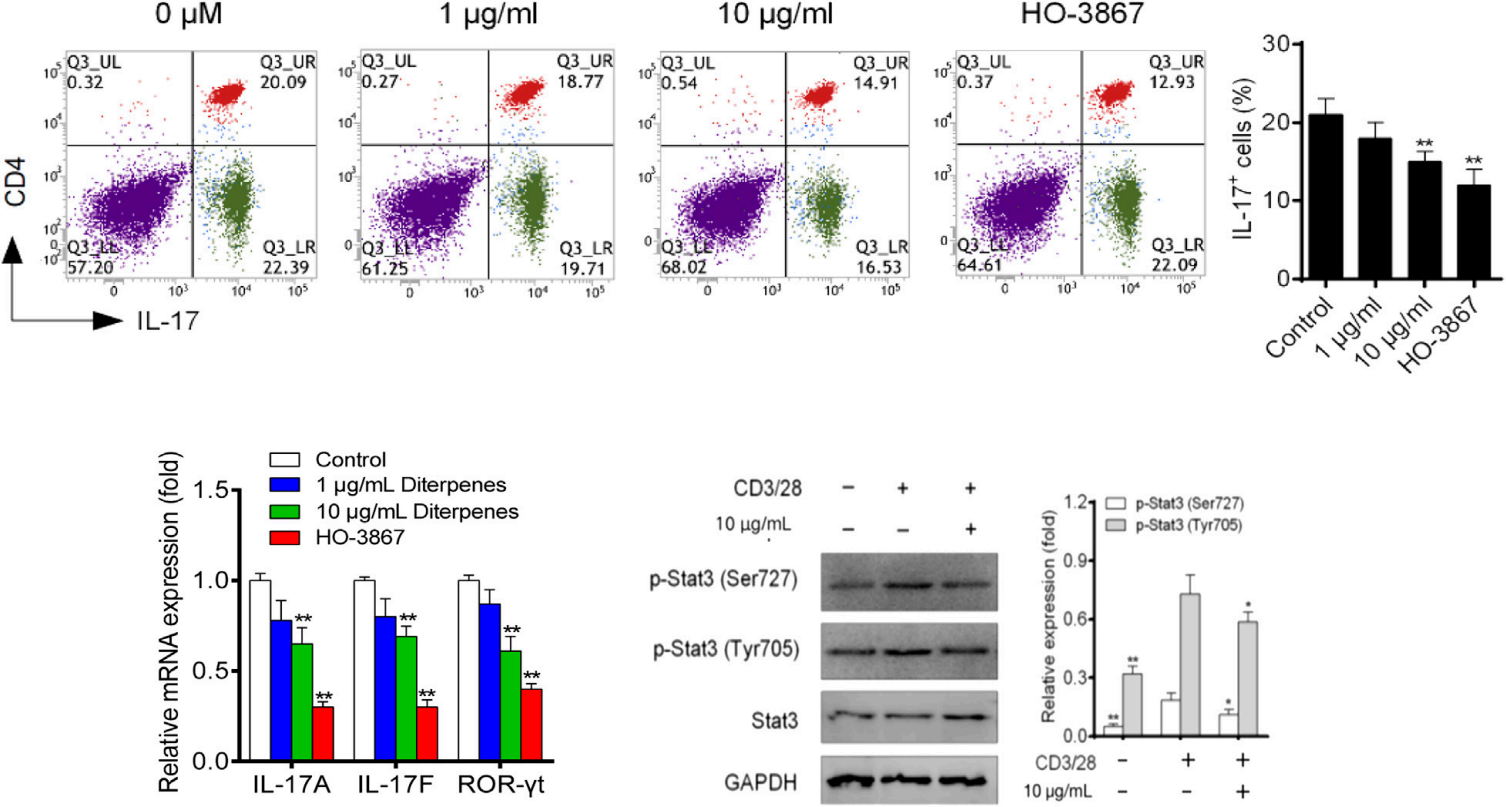
Effect of DF on the proportion of Th17 polarized cells, mRNA levels of Th17 transcription factors, and Th17-related STAT proteins. **, *p* < 0.05, compared with control group. GAPDH was used as a loading control.

Following culture under Th17 differentiation conditions for 4 days, approximately 30% of naive CD4^+^ T cells had polarized into Th17 cells, and DF addition inhibited this polarization in a dose-dependent manner (Figure 8). Th17 differentiation-related expression of IL-17a, IL-17f and ROR*γ*t was dose-dependently inhibited by DF (Figure 8). Furthermore, we found that 10 μg/ml of DF downregulated STAT3 phosphorylation, suggesting that DF inhibited Th17 differentiation by reducing STAT3 activation (Figure 8).

## 4 Discussion

To investigate the mechanism and related signaling pathways by which DF treats RA, this study systematically studied the role of DF in inducing Th cell polarization *in vivo* and *in vitro*.

The widely used *in vivo* model, LPS-induced mouse endotoxin shock, is employed *in vivo* to investigate the acute systemic inflammatory response (Agelaki et al., 2002). The results of the present study showed that DF could significantly improve the survival rate of the model mice (Figure 1); effectively alleviate LPS-induced endotoxin shock in the model mice; improve lung, liver, and kidney function; inhibit inflammatory cell infiltration; and reduce organ injury (Figure 2). It has been suggested that the imbalance of Th1/Th2 and Th17/Treg cells might be the cause of RA development (Boissier et al., 2008). The proinflammatory effects of Th1 and Th17 cells have been suggested to be involved in various inflammatory conditions in mice and humans, whereas Th2 and Treg cells have anti-inflammatory effects (Chen et al., 2012). In this study, splenic cell proliferation was inhibited by DF, which selectively inhibited Th1 and Th17 cells, and maintained the differentiation and function of Th2 and Treg cells. Pro-inflammatory cytokines, e.g., IL-6, IL-1, and TNF, orchestrate the inflammatory response (Sasaki et al., 2017). Constitutive overproduction of these pro-inflammatory cytokines is responsible for RA (Mohagheghpour et al., 2000; Kishimoto, 2010). In this experiment, DF at 0.2 mg/kg inhibited the secretion of TNF-α (*p* < 0.01), IL-1β (*p* < 0.01), IL-6 (*p* < 0.01), and other inflammatory cytokines (Figure 5).

Subsequently, the effects of DF on Th cell polarization and the JAK/STAT signaling pathway were studied. Assisted by *in situ* IFN-*γ*, IL-12 induction of STAT1/4 and the subsequent induction of the T-bet transcription factor are required for differentiation into Th1 cells (Mosmann and Coffman, 1989; Nakayamada et al., 2012). Under Th1-inducing conditions, the Th1 differentiation of STAT1-deficient (*STAT1* ^*−/−*^) naive CD4^+^ T cells is impaired and *STAT1* ^*−/−*^Th1 cells express less IFN-*γ* and T-bet (Ma et al., 2010). After activation by cytokines, the latent cytosolic factor STAT4 is phosphorylated, followed by its nuclear accumulation. Specific gene transcription is stimulated by activated STAT4, including IFNG (Remmers et al., 2007). To reduce the Th17 cell-related inflammatory response, many steps of Th17 cell differentiation or the secretion of IL-17 and other Th17-related cytokines or factors have been identified as possible targets (Boniface et al., 2013). Among them, ROR*γ*t, the Th17 cell characteristic transcription factor, participates in inflammatory responses and autoimmune diseases. Meanwhile, IL-17a and IL-17f have pro-inflammatory effects, and can induce the production of TNF and IL-6, which are the key cytokines promoting the aggregation, activation, and migration of neutrophils (Korn et al., 2009). STAT3 is activated by Thl7 cytokines and binds directly to *IL17A*, *IL17F*, and *IL2L* gene promoters (Huh and Littman, 2012). In the *in vitro* Th17 differentiation assay, STAT3 deficiency significantly weakened Thl7 cell differentiation, while overexpression of *STAT3* significantly upregulated IL-17 expression (Mathur et al., 2007). The results showed that DF inhibited the secretion of IFN-γ and IL-17 *in vivo* and their participation in the JAK/STAT signaling pathway, thus inhibiting the activation of STAT1 and STAT4. Moreover, DF inhibited the differentiation of Th1 and Th17 cells by decreasing the expression of T-bet and RORγt. Compared with IFN-γ and IL-17, DF had a weaker inhibitory effect on Treg type cytokine IL-10. Above all, DF maintained the differentiation and function of Th2 cells and Tregs, while inhibiting Th1 and Th17 cells. Moreover, the JAK/STAT signaling had an important function in DF’s regulation of Th cell polarization. These results demonstrated the potential therapeutic effect of DF on RA.

In the Th1 and Th17 cell polarization model, DF induced downregulation of the Th1 and Th17 cell ratio, and the expression levels of T-bet and RORγt were significantly inhibited after DF intervention. Furthermore, DF could significantly inhibit Th1-related cytokine IFN-γ and Th17-related cytokines IL-17a and IL-17f expression levels, and inhibit Th1 and Th17 polarization by decreasing the activation of STAT1, STAT4, and STAT3, respectively. In conclusion, we showed that DF has a marked immune regulatory role in the *in vitro* experiments, and the mechanism of action was basically the same as that *in vivo*. Through this study, the important function of JAK/STAT signaling in the anti-RA effect was clarified, providing the basis for further pharmacodynamic evaluation of DF.

## 5 Conclusion

*In vivo* and *in vitro* experiments demonstrated that DF could regulate Th cell polarization through the JAK/STAT signaling pathway, and could inhibit abnormally elevated immune responses by inhibiting Th1 and Th17-mediated immune responses, thus blocking the pathological process of RA. As a result, our findings might not only expand our understanding of the anti-RA properties of DF, but also provide a potential alternative for the prevention and treatment of RA.

## Data Availability

The original contributions presented in the study are included in the article/Supplementary Material, further inquiries can be directed to the corresponding authors.
