# Supplementary material for: The regulation of T helper cell polarization by the diterpenoid fraction of Rhododendron molle based on the JAK/STAT signaling pathway
Source: Front Pharmacol. 2022 Oct 25;13:1039441. doi: 10.3389/fphar.2022.1039441 (PMC9640628; doi:10.3389/fphar.2022.1039441)
Supplement: Supplementary file 1 [file DataSheet2.PDF]

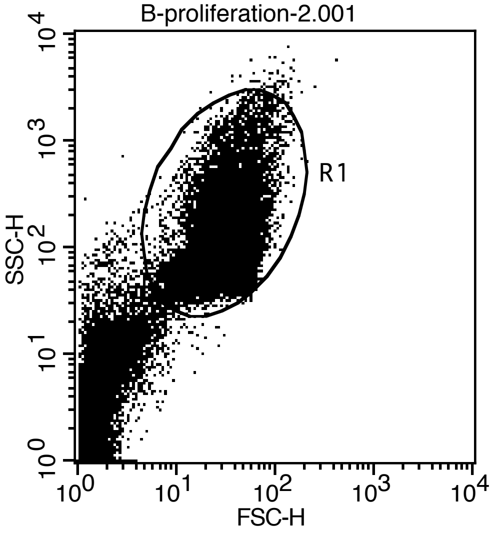

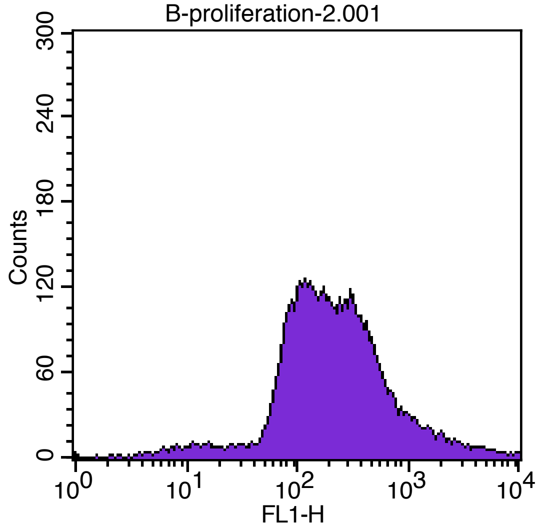

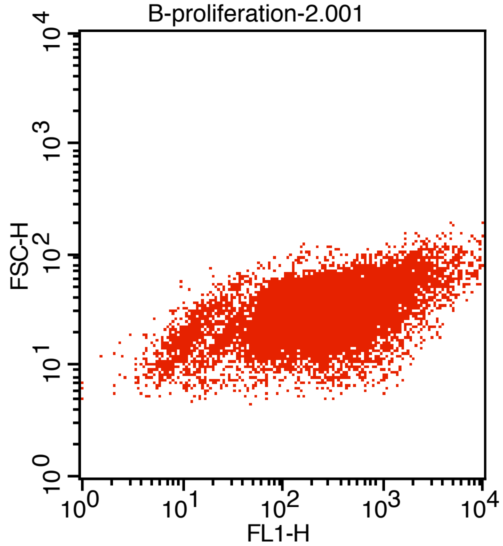

File: B-proliferation-2.001 Gate: G1

Marker	Events	% Gated	Mean
All	29462	100.00	370.56

File: B-proliferation-2.001

Region	Events	% Gated
R1	29462	100.00

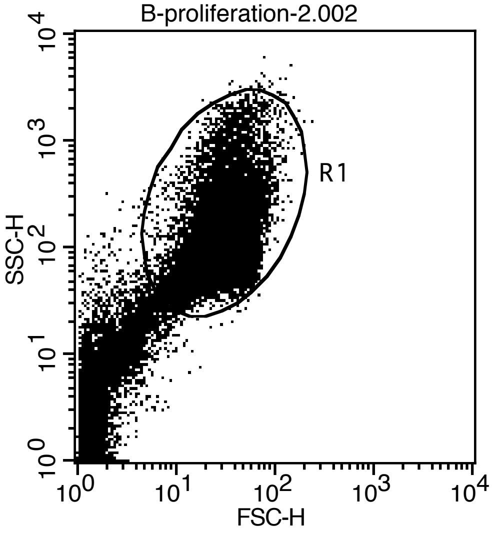

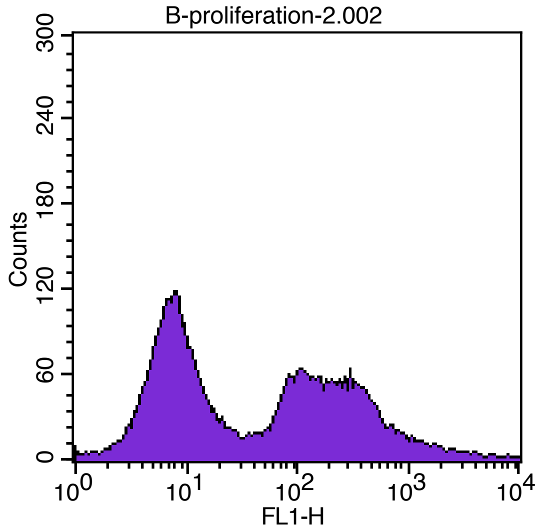

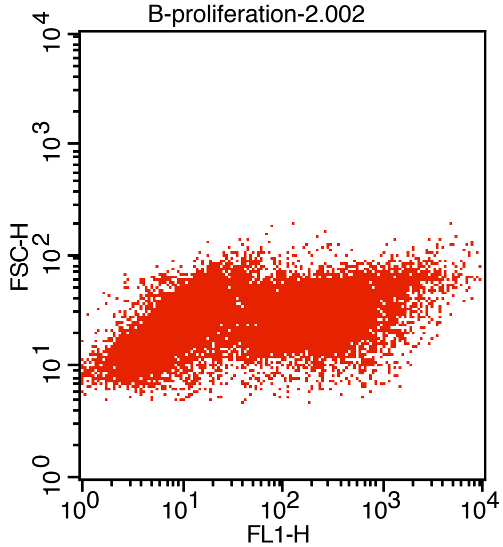

File: B-proliferation-2.002 Gate: G1

Marker	Events	% Gated	Mean
All	29370	100.00	183.66

File: B-proliferation-2.002

Region	Events	% Gated
R1	29370	100.00

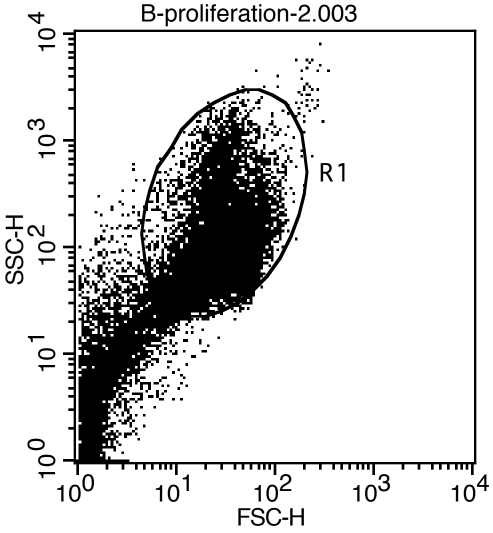

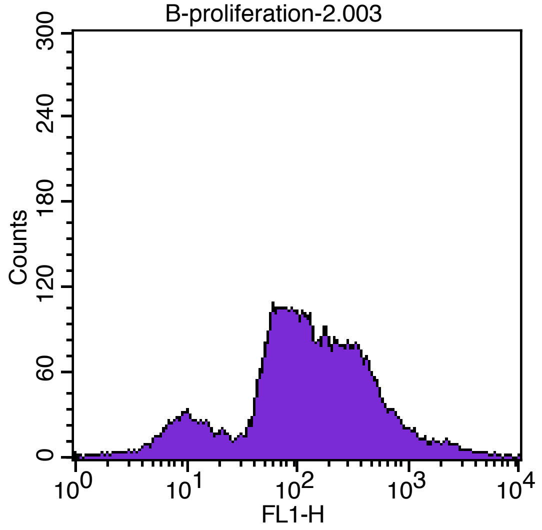

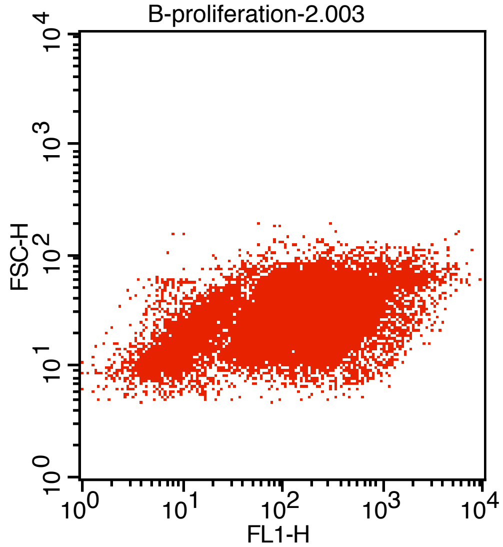

File: B-proliferation-2.003 Gate: G1

Marker	Events	% Gated	Mean
All	29190	100.00	248.23

File: B-proliferation-2.003

Region	Events	% Gated
R1	29190	100.00

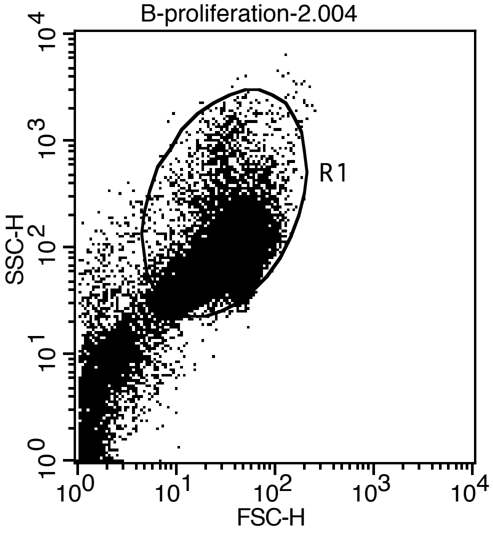

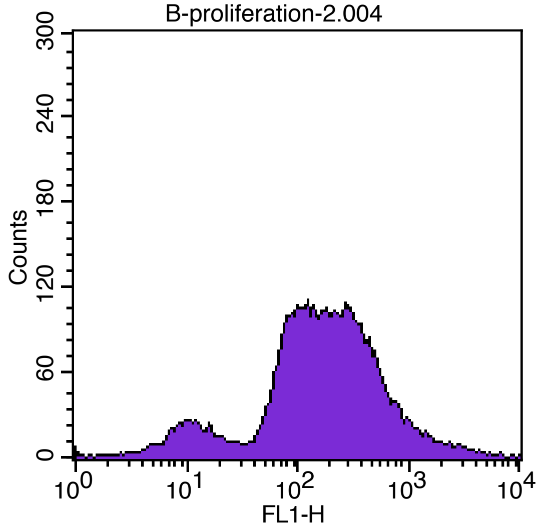

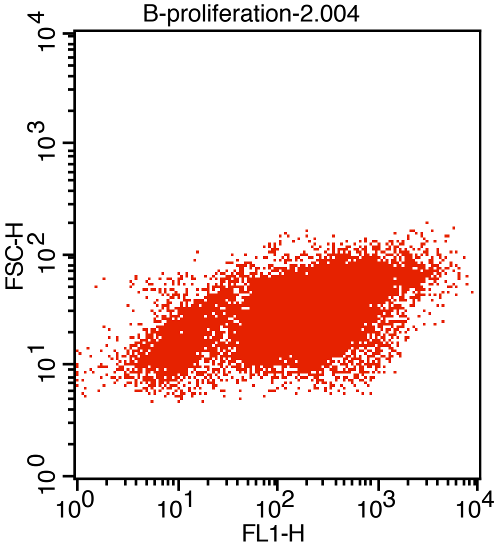

File: B-proliferation-2.004 Gate: G1

Marker	Events	% Gated	Mean
All	29282	100.00	291.53

File: B-proliferation-2.004

Region	Events	% Gated
R1	29282	100.00

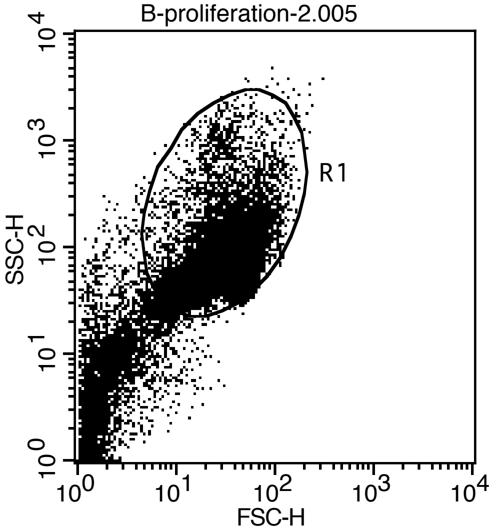

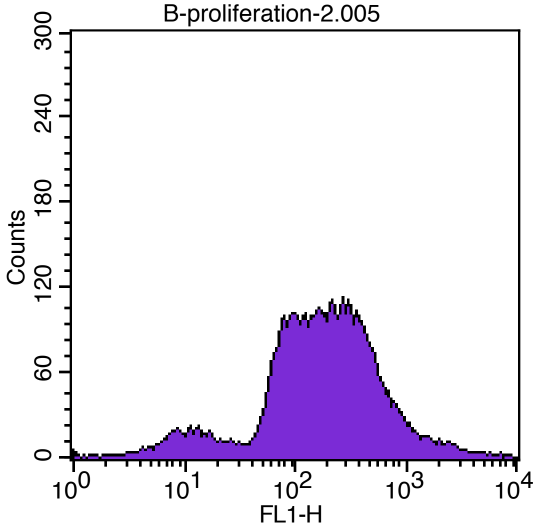

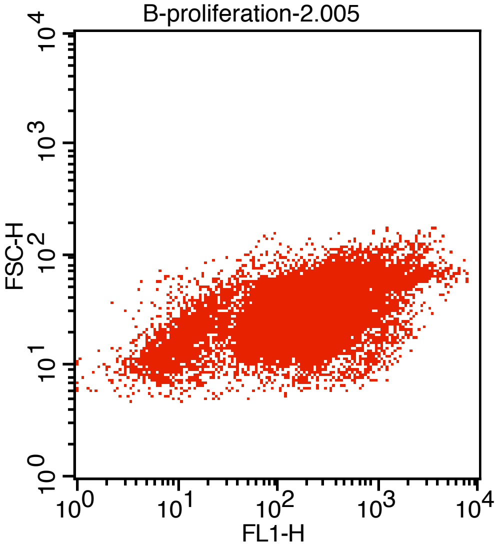

File: B-proliferation-2.005 Gate: G1

Marker	Events	% Gated	Mean
All	29305	100.00	295.81

File: B-proliferation-2.005

Region	Events	% Gated
R1	29305	100.00

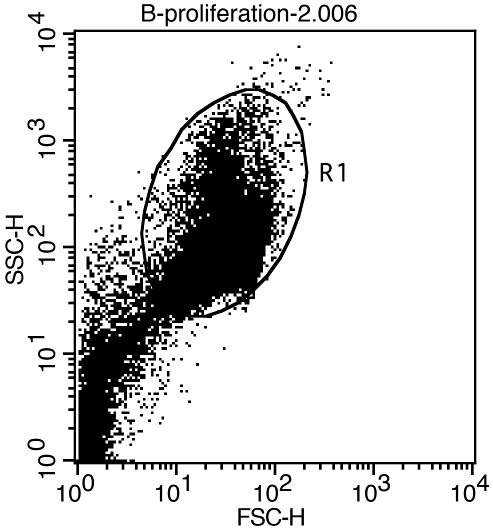

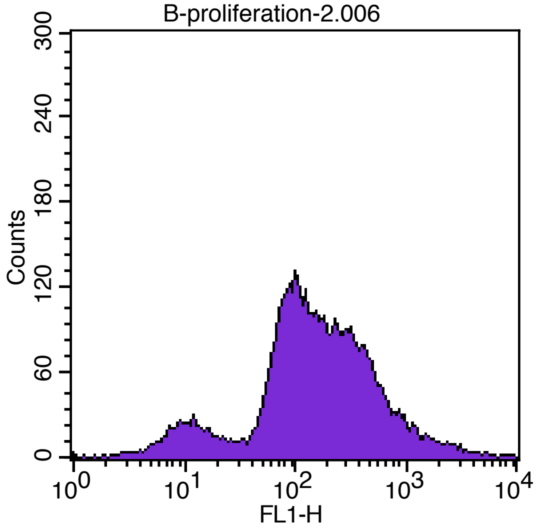

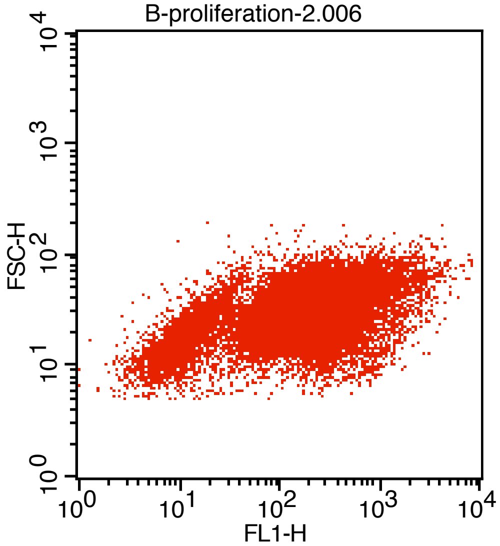

File: B-proliferation-2.006 Gate: G1

Marker	Events	% Gated	Mean
All	29403	100.00	276.51

File: B-proliferation-2.006

Region	Events	% Gated
R1	29403	100.00
