# Supplementary material for: The regulation of T helper cell polarization by the diterpenoid fraction of Rhododendron molle based on the JAK/STAT signaling pathway
Source: Front Pharmacol. 2022 Oct 25;13:1039441. doi: 10.3389/fphar.2022.1039441 (PMC9640628; doi:10.3389/fphar.2022.1039441)
Supplement: Supplementary file 2 [file DataSheet3.PDF]

TABLE 1 Sequences of the primers used in this study.

Gene	Forward Primers 5'-3'	Reverse Primers5'-3'
T-bet	CCTGGACCCAACTGTCAACT	AACTGTGTTCCCGAGGTGTC
IFNγ	TCTGAGACAATGAACGCTAC	TGGACCACTCGGATGAG
IL-17A	TCCAGAAGGCCCTCAGACTA	AGCATCTTCTCGACCCTGAA
IL-17F	GTGTTCCCAATGCCTCACTT	GTGCTTCTTCCTTGCCAGTC
RORγt	TGCAAGACTCATCGACAAGG	AGGGGATTCAACATCAGTGC
GAPDH	AGTGGCAAAGTGGAGATT	GTGGAGTCATACTGGAACA
